# DUOX-Mediated Signaling Is Not Required for LPS-Induced Neutrophilic Response in the Airways

**DOI:** 10.1371/journal.pone.0131810

**Published:** 2015-07-06

**Authors:** Sandra Chang, Angela Linderholm, Richart Harper

**Affiliations:** Department of Internal Medicine, Division of Pulmonary and Critical Care Medicine, School of Medicine, University of California at Davis, Davis, California 95616, United States; National Jewish Health, UNITED STATES

## Abstract

Oxidant production from DUOX1 has been proposed to lead to neutrophil recruitment into the airways when lung homeostasis is compromised. The objective of this study was to determine whether DUOX-derived hydrogen peroxide is required for LPS-induced neutrophil recruitment, using a functional DUOX knock out mouse model. We found that LPS induced profound neutrophilic lung inflammation in both Duoxa+/+ and Duoxa-/- mice between 3h and 24h. Duoxa-/- mice had significantly higher neutrophil influx 24h after LPS instillation despite similar cytokine levels (KC, MIP-2, or TGF-α) between the two groups. These findings suggest that LPS-TLR-4-induced KC or MIP-2 cytokine induction and subsequent neutrophil recruitment in the airway does not require DUOX-derived hydrogen peroxide from airway epithelium.

## Introduction

Dual oxidases (DUOX1 and DUOX2) are NADPH oxidases located at the epithelial surface of airway cells, and locally produce hydrogen peroxide (H_2_O_2_)[1]. The function of DUOX-mediated hydrogen peroxide production in the airway has been shown to provide direct lung host defense functions against Gram-negative[2] or Gram positive bacteria[3]. Additionally, increasing data suggest that DUOX-mediated H_2_O_2_ is responsible for oxidant-mediated signaling important for airway host defense.

Previously, several model systems have shown that H_2_O_2_ directly causes neutrophil chemotaxis[4–8] and several recent reports suggest that DUOX plays an important role in neutrophil chemotaxis in the airway in response to a variety of stimuli[9–11]. *In vitro* studies suggest that DUOX-derived H_2_O_2_ is essential for TGF-α signaling that subsequently leads to increased production of the neutrophil chemokine IL-8[10,11]. An *in vivo* murine model demonstrated that secretion of a mouse homolog of IL-8, MIP-2, is dependent upon DUOX2 activity[12]. And, we recently reported that DUOX is required for neutrophil recruitment in a mouse model of allergic asthma[9].

Collectively, these studies strongly implicate that DUOX regulates neutrophil chemotaxis through the canonical lipopolysaccharide-induced TLR4 signaling pathway, subsequently upregulating IL-8. Lipopolysaccharide (LPS) found on gram negative bacteria such as Pseudomonas aeruginosa was effective in stimulating DUOX activity, which potentially leads to neutrophil chemotaxis and wound repair[2,10,11,13]. Recently, an *in vivo* study by Li et al. found that diphenyleneiodonium chloride (DPI), a nonspecific NADPH oxidase inhibitor, suppressed neutrophil localization in bronchoalveolar lavage fluid after LPS exposure implicating DUOX as the key regulator of this recruitment[14].

It is firmly established that TLR4 is expressed on airway epithelium. However, the relative contribution of the airway epithelium versus hematopoietic cells in recruiting neutrophils after LPS challenge is less clear[15]. To better characterize the role of the airway epithelium, through DUOX-derived H_2_O_2_, to activate LPS-mediated neutrophil chemotaxis, we utilized a *Duoxa* ^*-/-*^ knockout mouse model that does not express functional DUOX1 or DUOX2[9,16]. DUOX1 and DUOX2 have both been implicated as having active roles in LPS-generated inflammatory signaling, thus a model system that is deficient in both DUOX isoforms was an important first step to determine the specific isoform mediating LPS-dependent signaling. Because DUOX isoforms are not expressed in hematopoietic cells, this model allowed us to specifically characterize the role of DUOX expressed in airway epithelial cells. In addition, this model excludes the possibility of one DUOX isoform compensating for the loss of the other.

We hypothesized that DUOX-derived hydrogen peroxide is necessary to signal neutrophil migration into the lungs following LPS exposure, and that lack of functional DUOX will result in reduced neutrophil chemotaxis into the lung.

## Materials and Methods

### Knockout mouse model

*Duoxa* ^*-/-*^ knockout mice were generated as described previously[16] and mice were obtained as a generous gift from Dr. Helmut Grasberger. All *in vivo* experiments were performed in accordance with the University of California at Davis Institutional Animal Care and Use Committee (IACUC). Mice utilized for our experiments were acquired through subsequent breeding of these breeding pairs at the UC Davis facility. Male mice, of 129Sv6 background, were maintained in HEPA-filtered laminar flow cage racks with a 12-hour light/dark cycle and allowed free access to food (Purina Rodent Chow) and water. Mice were housed and cared for by the veterinary staff of the UC Davis Animal Resource in AALAC-accredited facilities. Because *Duoxa* ^*-/-*^ mice are severely hypothyroid without hormone replacement[16], we supplemented mice with L-T4 hormone replacement as described previously[9]. Anesthesia and euthanasia procedures were performed according to UC Davis IACUC-approved protocols. All in vivo experiments were performed in accordance with the University of California at Davis Institutional Animal Care and Use Committee (IACUC) and specifically approved this study.

### LPS Exposure

LPS from *Pseudomonas aeruginosa* 10, source strain ATCC 27316 (Sigma-Aldrich L8643) was diluted with phosphate-buffered saline (PBS). Mice were anesthetized with isoflurane and 40uL of either PBS (control), or 1μg or 10μg LPS dissolved in PBS was administered via intratracheal instillation. LPS-exposed animals and PBS controls were necropsied at 3h, 6h, 12h, 24h, and 7 days after instillation.

### Bronchoalveolar lavage sample collection and processing

Mice were euthanized at specified timepoints with an intraperitioneal (IP) overdose of pentobarbital. The lungs were then lavaged two times with 1mL sterile PBS (pH = 7.4) to collect bronchoalveolar lavage fluid (BALF). BALF was centrifuged at 2000 rpm for 10 minutes and supernatant was collected and stored at -80°C. The resulting BALF cell pellet was resuspended in ACK/RBC lysis buffer and the pellet was resuspended in PBS. Live cell concentrations were estimated by counting trypan-blue-excluding cells on a hemacytometer. To determine BALF cell differentials, cytocentrifuge preparations were stained with a Hema3 kit as described in the manufacturer's instructions (Fisher Scientific, Kalamazoo, MI), and sealed using Cytoseal 60 (Richard-Allen Scientific, Kalamazoo, MI). Cell percent differentials were calculated by counting 10 fields at 400× magnification and classifying cell types as alveolar macrophage, neutrophil, eosinophil, lymphocyte, or “other” based upon standard morphological characteristics and staining profiles. Absolute cell counts were calculated by multiplying live cell counts by the cell type percent.

### Enzyme-Linked Immunosorbant Assay (ELISA) analyses

The supernatant fraction of the BALF was thawed on ice and used in enzyme-linked immunosorbant assays (ELISA). The mouse homologs of human interleukin (IL)-8, Keratinocyte-Derived Cytokine (KC) and Macrophage Inflammatory Protein (MIP)-2, were detected using ELISA (R&D Systems, Product Number MKC00B and MM200, respectively). TGF-α was also analyzed similarly (R&D Systems, Product number DTGA00). BALF cytokine concentrations were determined by comparison to standard curves for each cytokine provided by the supplier.

### Statistics

All data was processed using Prism 5 software (GraphPad Software, Inc., San Diego, California). Data was analyzed using 2-Way ANOVA followed by Bonferroni correction when appropriate. Data was deemed statistically significant at p ≤0.05.

## Results

### LPS Dose Response and Time Course

**Fig 1:**
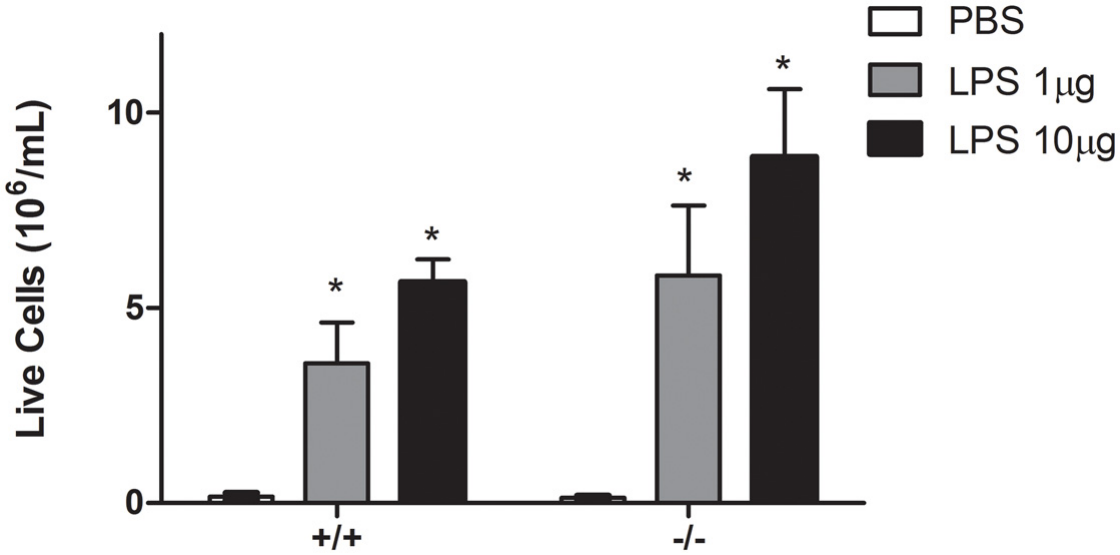
Dose response to LPS in *Duoxa* ^*+/+*^ and *Duoxa* ^*-/-*^ mice. Leukocytes were collected from the airway compartment by BAL 24 hours after intratrachael instillation of LPS (1μg or 10μg). The number of live cells was determined by trypan blue exclusion. Live cell counts are displayed for PBS control (open box), 1μg LPS (gray box), or 10μg LPS for both *Duoxa* ^*-/-*^ (-/-) and *Duoxa* ^*+/+*^ (+/+) mice. Data represent mean ± SEM from six animals in each group; * = p<0.05 compared to PBS control.

We evaluated live cell count dose responses to 1μg or 10μg LPS between *Duoxa* ^*-/-*^ and *Duoxa* ^*+/+*^ mice (Fig 1). Both *Duoxa* ^*-/-*^ and *Duoxa* ^*+/+*^ mice had robust increases in live cell counts after LPS instillation compared to PBS control. However, there appeared to be no dose response between the two doses of LPS we utilized. Given the lack of statistical significance in cell counts between the two doses, we utilized the 1μg dose of LPS for the remainder of our experiments.

**Fig 2:**
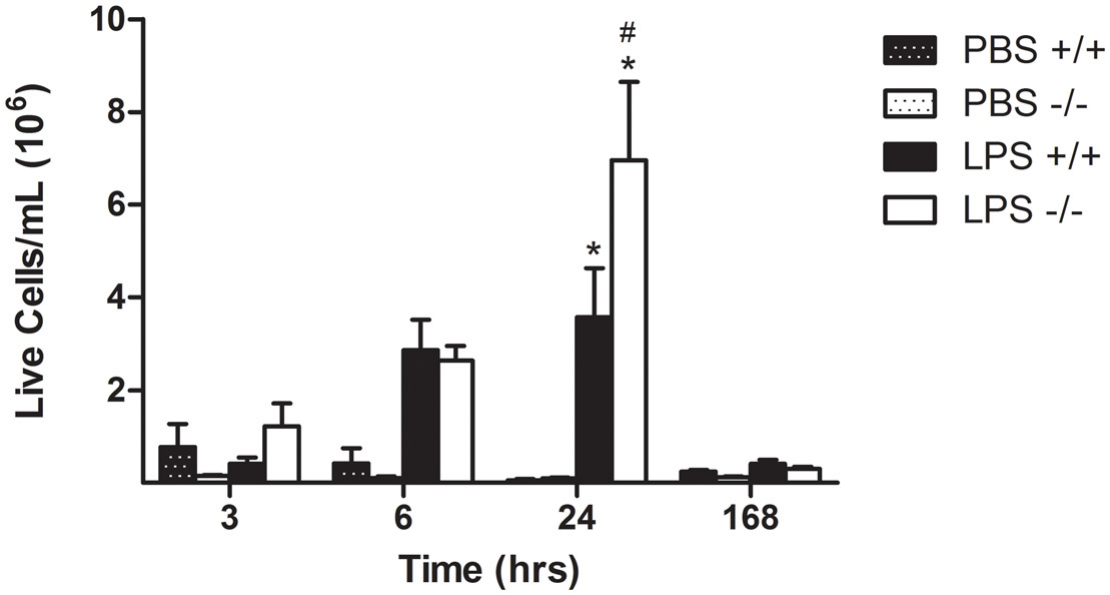
Time course of LPS-induced airway inflammation. Leukocytes were collected from the airway compartment by BAL at various timepoints up to 7 days (168h) after intratracheal instillation of 1μg LPS. The number of live cells was determined by trypan blue exclusion. Live cell counts are displayed for PBS control and LPS-exposed *Duoxa* ^*-/-*^ (-/-) and *Duoxa* ^*+/+*^ (+/+) mice as indicated. Data are shown as mean ± SEM for six mice in each group; * = p< 0.05 between LPS-treated and PBS-treated controls, # = p<0.05 between LPS-treated *Duoxa* ^*+/+*^ and *Duoxa* ^*-/-*^ mice.

To evaluate the LPS-induced influx of inflammatory cells in the lung over time, total live cell counts were analyzed and compared between *Duoxa* ^*-/-*^ and *Duoxa* ^*+/+*^ mice at 3h, 6h, 24h, and 7 days (Fig 2). Both *Duoxa* ^*-/-*^ and *Duoxa* ^*+/+*^ mice demonstrated increasing live cell counts at each timepoint up to 24h which subsided at 7 days. While *Duoxa* ^*-/-*^ and *Duoxa* ^*+/+*^ mice had similar trends in live cell counts for all timepoints, *Duoxa* ^*-/-*^ mice had significantly increased live cell counts at 24h when compared to *Duoxa* ^*+/+*^ mice (p ≤0.05).

### LPS-induced Neutrophil Influx

**Fig 3:**
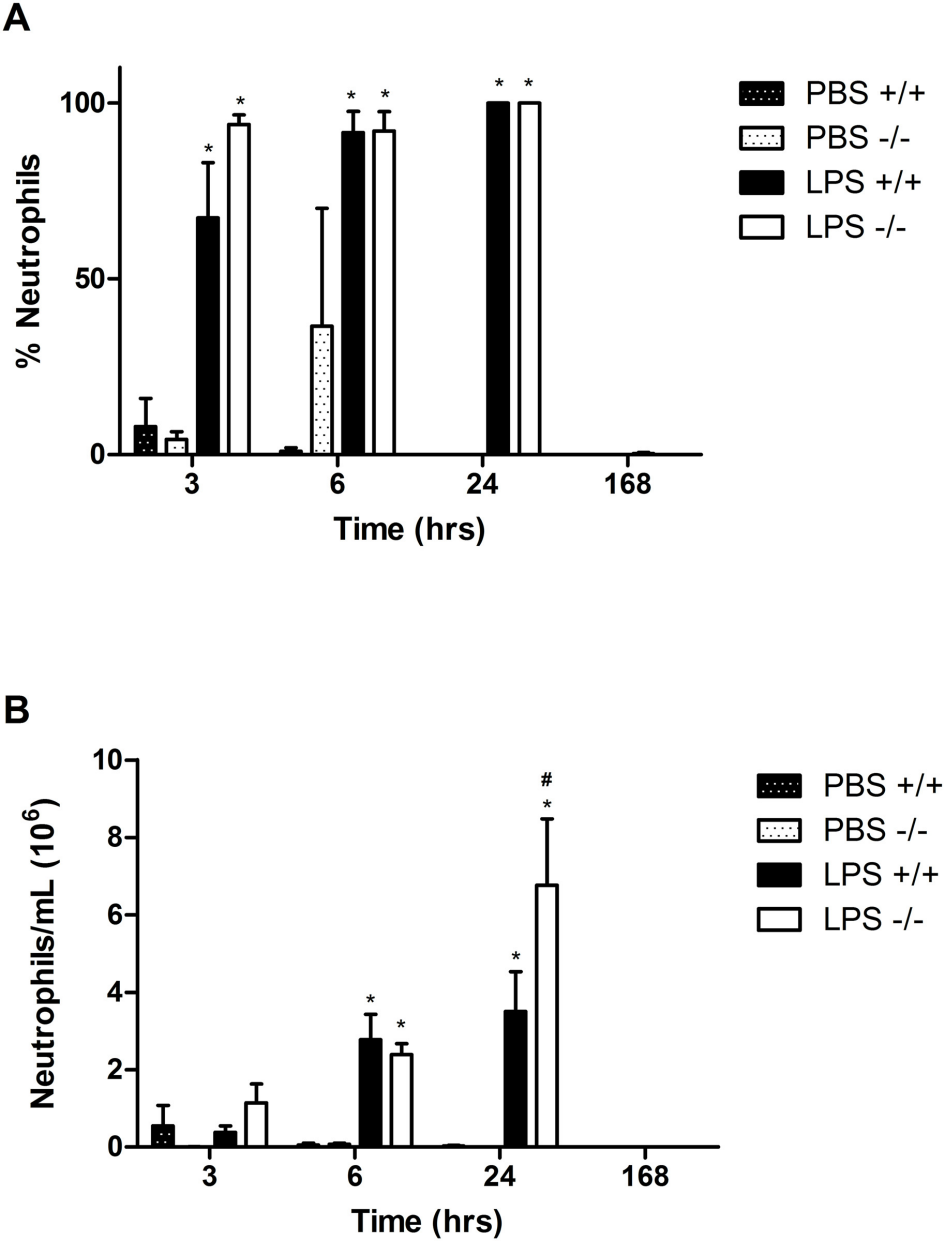
LPS induces predominantly neutrophilic inflammation in both *Duoxa* ^*-/-*^ and *Duoxa* ^*+/+*^ mice. Leukocytes were collected from the airway compartment by BAL at various timepoints up to 7 days (168h) after intratracheal instillation of 1μg LPS. Cell differentials were determined visually based on cell morphology and the percent of neutrophils (A) was compared between *Duoxa* ^*-/-*^ (-/-) and *Duoxa* ^*+/+*^ (+/+) mice. Absolute neutrophil counts (B) were calculated by multiplying neutrophil percentage with total cell number. Data are shown as mean±SEM from six mice in each group; * = p< 0.05 between LPS-treated and PBS-treated controls, # = p<0.05 between LPS-treated *Duoxa* ^*+/+*^ and *Duoxa* ^*-/-*^ mice.

We analyzed the BALF for macrophages, neutrophils, eosinophils and lymphocytes at 3h, 6h, and 24h after LPS exposure to determine the cell populations recruited by LPS in *Duoxa* ^*-/-*^ and *Duoxa* ^*+/+*^mice. As expected, we observed predominant neutrophilic inflammation in LPS-exposed *Duoxa* ^*+/+*^mice (Fig 3A). Surprisingly, *Duoxa* ^*-/-*^ mice had similar levels of neutrophilic inflammation after LPS exposure (Fig 3A), which conflicts previous reports. Similar to the live cell counts, both *Duoxa* ^*-/-*^ and *Duoxa* ^*+/+*^ mice demonstrated steadily increasing absolute neutrophils counts that peaked at 24h and subsided at 7 days. However, counter to what we would predict *a priori*, *Duoxa* ^*-/-*^ mice had a statistically significant increase in neutrophils at the 24h timepoint (Fig 3B).

Analysis of BALF seven days after LPS exposure demonstrated a return to a macrophage-predominant cell profile with a slightly elevated lymphocyte population in both *Duoxa* ^*-/-*^ and *Duoxa* ^*+/+*^mice with no significant differences between the two groups of animals (data not shown).

### LPS-induced Cytokine Production

**Fig 4:**
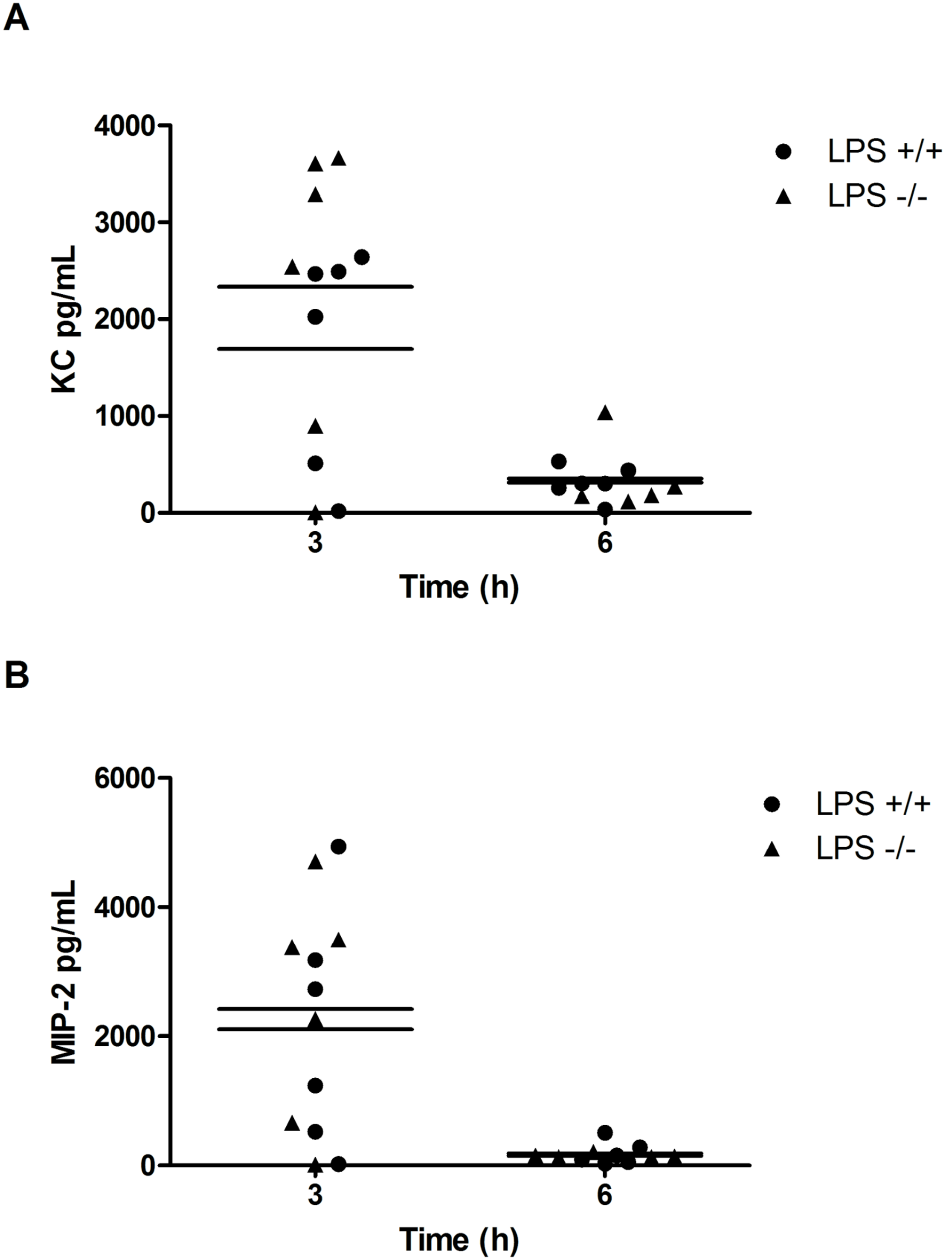
Cytokine levels are similar between LPS-exposed *Duoxa* ^+/+^ and *Duoxa*-^/-^ mice. BALF was collected at various timepoints from *Duoxa* ^+/+^ (+/+) and *Duoxa*-^/-^ (-/-) mice followed by measurement of KC (A) or MIP-2 (B) cytokine levels in the supernatant by ELISA. Cytokine concentration was determined by comparison to standard controls for each cytokine. Data from six animals in each group are shown. Mean cytokine values from *Duoxa* ^+/+^ (+/+) and *Duoxa*-^/-^ (-/-) mice are shown as a vertical line. Cytokine levels for LPS treatment differed significantly than PBS controls at three hours in both groups of mice (data not shown).

Typically, the binding of LPS to the TLR-4 receptor activates a signaling cascade that leads to increased IL-8 production and subsequent neutrophil recruitment[17], and DUOX-derived hydrogen peroxide has been shown to play a role in LPS-induced IL-8 production[10,11,13]. Therefore, we measured changes in the IL-8 mouse homologs KC and MIP-2[18] in BALF from *Duoxa* ^*-/-*^ and *Duoxa* ^*+/+*^ mice after LPS instillation to evaluate the impact of DUOX-derived hydrogen peroxide in neutrophil chemotaxis. Both KC and MIP-2 levels peaked at 3h consistent with the canonical LPS-TLR4-IL-8 signaling pathway. Surprisingly, LPS induced similar levels of IL-8 homologues in the *Duoxa* ^*-/-*^ mice compared with the *Duoxa* ^*+/+*^ mice (Fig 4). Alternatively, previous studies suggested LPS initiates DUOX-dependent upregulation of TGF-α signaling in airway epithelium, which may be primarily responsible for neutrophil influx into the airway[10,11,13,14]. To evaluate this possibility, we compared TGF-α levels in BALF from *Duoxa* ^*-/-*^ and *Duoxa* ^*+/+*^ mice and found no induction of TGF-α in *Duoxa* ^*-/-*^ or *Duoxa* ^*+/+*^ mice (data not shown). This supported our observation that LPS-induced neutrophil recruitment occurred through modulated expression of KC and MIP-2 independent of TGF-α signaling.

## Discussion

Intratracheal LPS administration is known to recruit neutrophils into the lungs of mice[17,19,20]. This occurs through LPS serving as a ligand for TLR-4, subsequently activating transcription of the major neutrophil chemokine IL-8[21] in humans, or KC and MIP-2, mouse homologs of IL-8, in mice[18,22]. We previously identified DUOX as a major source of epithelial-derived H_2_O_2_ in isolated murine epithelium, and an important enzyme for neutrophil recruitment in a mouse model of allergic asthma. [9]. Based on these results and the existing literature, we hypothesized that DUOX-derived hydrogen peroxide would act as an important signaling molecule for neutrophil migration into the lungs following LPS exposure. Surprisingly, our data suggest that LPS-mediated neutrophil recruitment in the mouse lung does not require DUOX-dependent H_2_O_2_ signaling.

H_2_O_2_, the primary product of DUOX enzymatic activity, is known to be a critical factor for multiple intracellular signaling pathways[23,24]. In this report, we investigated the role of DUOX-generated H_2_O_2_ in LPS-mediated neutrophil recruitment to the lung. Previously, other groups have demonstrated associative correlations between DUOX and LPS-mediated neutrophil signaling through either IL-8 or TGF-α[10,11,14], and we investigated both possibilities in our model.

Nakanaga et al. characterized DUOX-dependent LPS signaling in NCI-H292 cells, a human pulmonary mucoepidermoid carcinoma cell line. They demonstrated that DUOX-derived hydrogen peroxide activated TGF-α converting enzyme (TACE), followed by TGF-α release, binding to epidermal growth factor receptor (EGFR), and subsequent increases in IL-8 production *in vitro*[10]. Similarly, Boots et al., utilizing HBE1 cells, an immortalized human bronchial epithelial cell line, found that DUOX-generated H_2_O_2_ was necessary for TGF-α-mediated constitutive EGFR activity and increased IL-8 production[11]. In contrast, we did not observe any differences in TGF-α, KC, or MIP-2 between *Duoxa* ^*-/-*^ or *Duoxa* ^*+/+*^ mice after LPS exposure (Fig 4 and data not shown). Our data suggest that LPS is able to signal neutrophils through increased KC or MIP-2 independent of either DUOX isoform. Additionally, TGF-α had no apparent role in LPS-induced neutrophil recruitment.

Because these previous studies were done in cell culture models, it is possible that our conflicting findings are due to compensatory mechanisms available in the *in vivo* system that do not occur during short-term DUOX inhibition in cell culture systems, the use of alternative NOX isoforms *in vivo*, or both [25,26]. We did not observe compensatory increases in NOX4, the most likely alternative NOX to be expressed in the absence of DUOX expression[26], in the airway epithelium of these mice (data not shown), but we cannot exclude an unusual upregulation of other NOX isoforms.

Alternatively, the specific stimulus may be the primary difference. Although there is clear evidence that DUOX is required for neutrophil recruitment in multiple model systems[7,9,14,27], i*n vivo* models that evaluate mechanisms of DUOX-mediated neutrophil recruitment in the airway are limited. Li et al. reported that LPS-induced neutrophil chemotaxis into the lung of mice was reduced with the general NADPH oxidase inhibitor DPI[28,29]. Because DPI is relatively non-specific for all flavin proteins, these results may not account for potential alternative NOX(s) that are functioning in lieu of DUOX[30,31]. Similarly, Ryu et al. demonstrated that TLR-4 signaling induced features of allergic asthma that were dependent upon murine DUOX2-generated reactive oxygen species[32], but their studies did not exclude TLR-4-independent pathways that may be primarily responsible for DUOX-dependent neutrophil recruitment. Here, we specifically evaluated how LPS signals through DUOX *in vivo*, but directly demonstrated that neither DUOX isoform is required for LPS-mediated neutrophil recruitment. Together, our data suggest that DUOX is not required for LPS-TLR-4-dependent neutrophil recruitment, but is required for allergy-induced neutrophil recruitment.

We speculate that our observations are due to differences in the primary source of neutrophil recruitment. A primary mechanism for LPS-induced neutrophil recruitment in the lung involves interactions between neutrophils, vascular endothelial cells, and alveolar macrophages [33,34], which predominantly express Nox1 or Nox2. Mechanisms for ovalbumin-induced neutrophilic inflammation are not as well characterized, but potentially rely on airway specific proteins such as TNF-related apoptosis inducing ligand (TRAIL) [35–37]. Nonspecific inhibition of Nox proteins subsequently will inhibit both LPS- and OVA-induced neutrophil recruitment, whereas selective inhibition of DUOX proteins will only effect OVA-induced, or airway epithelium-dependent, mechanisms of neutrophilic inflammation. Further investigation of these differences potentially will reveal important novel pathways of neutrophil recruitment.

Surprisingly, we observed significantly more neutrophils recruited into the airway in *Duoxa* ^*-/-*^ mice compared to *Duoxa* ^*+/+*^ mice. These results suggest that LPS-induced neutrophil influx is enhanced in the absence of functional DUOX, in contrast to ova-induced neutrophil influx, where DUOX is required for neutrophil recruitment[9]. Because ROS are known regulators of cell differentiation[38], and DUOX expression has been found to increase with age in the developing lung[39,40], it is possible that the *Duoxa* ^*-/-*^ mice have impaired epithelial structural integrity or repair mechanisms, skewed epithelial cell populations, or altered cell-cell junctions to explain this observation. For example, neutrophils are known to cause lung epithelial damage during extravasation into the airway[41,42] and DUOX-derived hydrogen peroxide is crucial in lung epithelial repair and wound closure[13,43–46]. Without an intact DUOX-mediated repair mechanism, this “leaky” epithelium may allow an increased number of neutrophils to migrate from the blood vessels to the airways after LPS-triggered signaling.

## Conclusion

In contrast to previous studies, we have demonstrated that DUOX does not contribute specifically to LPS-mediated neutrophil recruitment. These differences may be due to compensatory mechanisms that occur in the long-term absence of both DUOX isoforms. Importantly, there is strong evidence that DUOX is important in allergy-induced neutrophilic inflammation[9,32] and future studies exploring these contrasting findings will likely reveal novel mechanisms of allergy-induced neutrophil influx.

## Supporting Information

S1 File ARRIVE checklist. (PDF)
